# Branching Out: A Retrospective Review of Tree Fall-Related Trauma

**DOI:** 10.7759/cureus.58136

**Published:** 2024-04-12

**Authors:** Kyle Nugent, Andrew McCague, Austin Henken-Siefken

**Affiliations:** 1 Surgery, Desert Regional Medical Center, Palm Springs, USA; 2 Trauma and Acute Care Surgery, Desert Regional Medical Center, Palm Springs, USA

**Keywords:** trees, trauma, falls from trees, falls from heights, falls

## Abstract

Introduction

Falls from trees (FFTs), although rare, represent a significant public health concern due to the severe consequences they can impose. Such incidents, while statistically uncommon across the wider population, have the potential to cause drastic, lasting alterations in patients’ lives. The severity of these events is often substantial, highlighted by high Injury Severity Scores (ISSs) and prolonged hospital length of stay (LOS), which brings to light the urgent need for preventive strategies and heightened awareness. Our study aims to present a current epidemiological understanding of the patterns, nature, and severity of injuries caused by FFTs. Additionally, it provides an analysis and comparison of data obtained from a de-identified trauma database of patients presenting after FFTs.

Methods

This review presents data from a trauma registry system detailing trauma admissions from March 31, 2016, to December 27, 2021, at the Desert Regional Medical Center in Palm Springs, California, United States, a designated Level 1 trauma center. Throughout this period of nearly five years and eight months, a total of 3,148 patients were recorded to have visited the emergency department due to falls. Specifically, the study zeroes in on the subset of patients who were admitted after experiencing FFTs. From the comprehensive retrospective examination, it was noted that among the 3,148 fall incidents, there were 50 cases that involved FFTs.

Results

This retrospective analysis focused on 50 patients treated at the emergency department after FFTs, with a predominantly male demographic profile of 49 (98%) and an average age of 44 years. Hospitalization was required for the vast majority (44%), with approximately one-third necessitating ICU care. Surgical procedures were necessary for 35 (70%) of these cases. Upon discharge, 36 (72% of patients) were able to return home. Vertebral fractures were the most frequent injury, present in 24 (22% of admissions), followed closely by soft tissue injuries at 23 (21%). The mean ISS was 11, although those with extended hospital stays of over 10 days had higher ISS scores of 16, in contrast to an ISS of 10 for those with shorter stays.

Conclusions

FFTs constitute a lesser-known category of trauma-related injuries in the broader spectrum of fall-related incidents. Although relatively infrequent, these incidents result in significant injury burdens. The objective of this review is to compile and summarize the existing body of literature on FFTs. It involves an in-depth analysis of admission, discharge, and demographic data related to FFTs, highlighting the significant consequences associated with such accidents. Additionally, this review incorporates an analysis of a specialized dataset dedicated to injuries resulting from FFTs, facilitating a comparative assessment against current research in this field.

## Introduction

Falls, especially those from trees, pose a significant risk to individuals of all ages. In the United States, falls are consistently cited as a primary cause of injury across nearly all age groups, indicating their widespread impact [1]. Specifically, falls from trees (FFTs) are associated with a range of injuries, from minor bruises to more severe outcomes like fractures or head trauma. While FFTs pose a significant risk in professions such as tree care, forestry, and farming, they are comparatively uncommon in the general population. A prospective study involving 60 patients who fell from trees highlighted the common occupational hazards faced by traditional farmers, resulting in severe and frequently multiple injuries. FFTs often lead to spinal injuries, becoming a significant contributor to traumatic quadriplegia and paraplegia [2]. With regard to the rarity of FFTs in the general population, a study from 2022 covering both rural and urban areas reported that out of 13,884 hospital admissions over a span of 8.5 years, only 37 were related to incidents involving trees [3]. While FFTs are infrequent, when they do happen, they typically result in multiple concurrent injuries, many of which can be severely disabling or life-threatening.

## Materials and methods

Our retrospective study analyzed de-identified trauma admission records from March 2016 to December 2021 at the Desert Regional Medical Center, a Level 1 trauma center located in Palm Springs, California, United States. The focus was on individuals who were injured due to FFTs. Falls were defined per the World Health Organization’s criteria as “an event which results in a person coming to rest inadvertently on the ground or floor or other lower level.” The inclusion criteria consisted of patients aged 1 and above who suffered FFTs while in an outpatient setting. Exclusion criteria encompassed individuals who fell from heights other than trees, such as roofs, ledges, ladders, and other non-tree structures. De-identified patient data, encompassing demographic details, Injury Severity Score (ISS), injury types, hospital length of stay (LOS), and patient outcomes, was retrieved from the electronic health records database of the Desert Regional Medical Center. The collected data were compiled into a Microsoft Excel spreadsheet (Microsoft Corporation, Redmond, Washington, United States) for analysis. Descriptive statistical techniques were employed to provide a concise overview of the dataset. This involved calculating mean values and standard deviations to understand the central tendency and variability of the data, respectively. Additionally, p-values were computed to assess the statistical significance of observed differences or associations. The statistical analysis aimed to summarize key findings regarding demographic characteristics, injury severity, hospital LOS, and patient outcomes among individuals injured due to FFTs.

## Results

Our dataset from the Desert Regional Medical Center, categorized as a Level 1 trauma facility in Palm Springs, California, United States, records the treatment of 50 outpatient FFTs from March 31, 2016, to December 27, 2021. Of these patients, there was a male predominance, with 49 (98%) males and one (2%) female. The age range included four (8%) patients 65 years of age or older. A large majority, 48 (96%), identified as Hispanic or Latino (Table 1). Emergency room visits led to hospital admission in 44 (88%) of the cases, with 14 (28%) of these requiring intensive care. Surgical procedures were performed on 35 (70%) of those admitted. There was one death reported within this group (Table 2). The average LOS at the hospital was 6.9 days and 5.2 days in the ICU (Table 3). The mean age at admission was 44 years, compared to 38.8 years for those not admitted (Table 4). The average ISS of those ≥65 was 8, and the average ISS of those <65 was 11 (Table 5). The average ISS was 11, with patients staying 10 days or less having an ISS of 10, and those staying longer than 10 days having an ISS of 16 (Table 6). Upon discharge, 36 (72%) returned home (Table 7). Vertebral fractures were the most common injury, present in 24 (22% of admissions), whereas lower extremity fractures occurred in 6 (5%) of cases (Table 8).

**Table 1:** Demographic data

Demographic characteristics	n (%)
Gender	
Male	49 (98%)
Female	1 (2%)
Age >65	4 (8%)
Age <65	46 (92%)
Race	
Hispanic or Latino	48 (96%)
Not Hispanic or Latino	2 (4%)

**Table 2:** Admission data

Disposition	Number of patients	Percentage
Admissions	44	88%
ICU admissions	14	28%
Surgery	35	70%
Death	1	2%

**Table 3:** LOS LOS, length of stay

	LOS (days)	SD (days)
Average LOS of admitted patients	6.9	7.4
Average LOS in the ICU	5.2	5.7

**Table 4:** Average age on admission versus no admission

	Average age (years)	SD (years)	p-value
Admitted	44	15.7	0.23
Not admitted	38.8	17.4	

**Table 5:** Injury severity by age ISS, Injury Severity Score

Age (years)	ISS score	SD	p-value
≥65	8	2.7	0.2
<65	10.95	6.8	

**Table 6:** Average ISS by LOS Note: * represents a significant p-value. ISS, Injury Severity Score; LOS, length of stay

	Average ISS	SD (ISS)	p-value
ISS score (all patients)	11	6	0.009*
ISS score with LOS ≤10 days	10	6	
ISS score with LOS >10 days	16	8	

**Table 7:** Discharge data ARU, acute rehabilitation unit; SNF, skilled nursing facility

Disposition	Number of patients	Percentage
Home	36	72%
ARU	6	12%
Unreported	5	10%
SNF	2	4%
Morgue	1	2%

**Table 8:** Presenting injuries on admission Total number of injury types (n) = 111 Note: We have categorized abrasions, contusions, strains, and sprains as soft tissue injuries. Additionally, subdural hematomas, subarachnoid hemorrhages, and concussions have been classified as TBIs. TBIs, traumatic brain injuries

Injury type	n (%)
Vertebral fracture	24 (22%)
Soft tissue injury	23 (21%)
Upper extremity fracture	12 (11%)
Rib fracture	12 (11%)
TBIs	12 (11%)
Laceration	11 (10%)
Hip/femur fracture	11 (10%)
Lower extremity fracture	6 (5%)

## Discussion

Our research findings indicate that individuals admitted after experiencing an FFT have an average age of 44 years, with a notable predominance of male patients. This contrasts with a 2022 study that highlighted that the most affected age group for unintentional tree failures, including FFTs, typically falls within the age range of 50-74 years. Moreover, the mentioned study pointed out that a significant proportion of these admissions, specifically 79.4%, comprised male patients [3]. The reasons behind these age and gender patterns are likely multifaceted and may be influenced by various factors, including engagement in outdoor activities, occupations related to tree work, and potential differences in risk perception and behavior among different age and gender cohorts.

The admission data further demonstrates that a significant proportion of patients required hospitalization, and among these hospitalized cases, nearly three-quarters necessitated surgical intervention. While existing research on hospital admissions and surgical procedures following FFTs is somewhat scarce, comprehensive data regarding falls from heights is readily accessible. For instance, a retrospective study conducted in 2018 examined 460 patients who fell from an average height of 1.1-4 meters. Among them, half received treatment in the emergency department and were subsequently discharged, while the remaining individuals were referred to either neurosurgery or orthopedics [4]. A significant percentage of falls from heights, including FFTs, require surgery due to the severity and nature of the injuries sustained during such incidents. Falls from significant heights often result in complex fractures of bones, such as the spine and pelvis, or long bones like the femur. Surgical procedures are often necessary to realign and stabilize these fractures [5]. Traumatic brain injuries, including intracranial bleeding and skull fractures, are common in falls from heights. Surgery may be required to relieve pressure on the brain, remove blood clots, or repair skull fractures [6].

Our findings from the data revealed that vertebral fractures and soft tissue injuries were the two most commonly diagnosed injuries upon admission. Vertebral compression fractures following falls are relatively common, comprising a significant portion of spinal fractures. Studies indicate that approximately 58% of all spinal fractures occur as a result of falls from standing height, highlighting their prevalence [7]. With regard to falls from heights, a 2021 study reports a 15.3% incidence of spine injuries in falls from great heights, highlighting the risk of sustaining severe thoracic injuries after such falls [8]. Vertebral compression fractures are common in falls from heights due to the significant axial or compressive forces exerted on the spine upon impact. When a person falls from a height, such as a standing position or higher, the force of impact is transmitted through the spine, leading to biomechanical failure of the vertebrae. The vertebrae may not be able to withstand this sudden and intense pressure, resulting in compression fractures [9]. Of note, aging is identified as a crucial factor in the prevalence of vertebral compression fractures, with the condition becoming more prevalent as individuals age, especially in women over 80 years old [10].

It is important to emphasize that the majority of the patients in our dataset presented with a combination of various injury types. These polytraumatic injuries, characterized by severe injuries affecting different parts of the body, are a significant concern following falls from heights. Among the polytrauma cases we observed, soft tissue injuries were particularly prevalent and were observed in nearly every patient. These injuries, including contusions, abrasions, and deep tissue damage resulting from blunt trauma, can potentially lead to complications such as infections and delayed wound healing. While not covered in our dataset, it is worth noting that psychological trauma, including conditions like post-traumatic stress disorder (PTSD), anxiety, and depression, can also be a potential outcome of FFTs and falls in general. Studies highlight that older adults who experience serious falls may develop symptoms of PTSD in the aftermath, with psychological impacts observed in the days following the event. Additionally, falls can trigger a fear of falling, anxiety, and depression, further exacerbating psychological distress [11].

Following hospital discharge, approximately 36 (72% of patients) were permitted to return to their residences for recovery, while around 6 (10%) were directed to an acute rehabilitation unit. This observation can be partially explained by the relatively youthful average age of admitted patients, 44 years. This demographic tends to exhibit a greater potential for swift recuperation and a higher degree of self-healing capability compared to older patients [12]. While there is no published data specifically on discharge information for patients after FFTs, a 2008 study focusing on hip fracture discharge locations sheds light on the significance of age in determining discharge destinations for hip fracture patients. Notably, younger patients, typically those under the age of 60, are less frequently discharged to alternative locations like rehabilitation facilities or nursing homes. The decision to discharge to home is influenced not only by younger age but also by the patient’s capacity to independently perform activities of daily living. Younger patients often possess better functional abilities, making it more feasible for them to return home following treatment for a fracture [13].

Research suggests that younger individuals typically exhibit lower ISS, indicating less severe injuries [14]. This correlation stems from factors such as physiological resilience and accelerated healing processes. Younger people often possess increased bone density and more flexible tissues, reducing the severity of injuries even in comparable accidents. Additionally, their bodies tend to recover faster from injuries, potentially mitigating long-term impacts and resulting in a lower ISS [15]. However, our study revealed a counterintuitive finding: individuals over 65 had lower average ISS scores compared to those under 65. This anomaly may be attributed to the disproportionate representation of younger patients in the dataset, with 92% falling below 65 years old.

Analysis of the dataset also revealed a clear connection between a higher ISS and an extended hospital LOS. This observation aligns with the results of a 2020 study investigating factors influencing hospital stay duration among trauma patients. The study emphasized the ISS as an effective predictor of hospital LOS and established a positive correlation between higher ISS scores and prolonged hospitalization [16]. The rationale behind this correlation is straightforward: as injury severity, as measured by ISS, increases, it typically necessitates more comprehensive medical interventions, including surgeries and rehabilitation. Consequently, patients with more severe injuries tend to experience an extended period of hospitalization due to the intensified care and recovery requirements. In summary, healthcare providers should take the ISS into careful consideration when assessing and strategizing the duration of hospital stays for trauma patients. This evaluation is crucial for effective resource allocation and optimizing patient care management.

It is important to recognize the limitations of our research. The relatively small sample size restricts our ability to observe infrequent events or variations that may become evident in a larger pool of data. Furthermore, the absence of data on the heights from which individuals fell limits our understanding of how the distance of a fall may impact the severity of the injuries incurred.

## Conclusions

FFTs represent a relatively uncommon category of falls, yet they carry a significant risk of injury for those involved. Our research findings have shown that individuals most frequently affected by FFTs tend to be in their 40s and predominantly male. The prevalent injuries associated with FFTs often include vertebral fractures and concomitant soft tissue damage. A substantial portion of patients require surgical intervention as part of their treatment, and the most common discharge location after treatment is the patient’s home. Additionally, it was noted that patients with a higher admitting ISS experienced extended hospital LOS. Despite FFTs being relatively rare, it is crucial for individuals of all age groups to acknowledge the inherent risks associated with elevated heights and the potential injuries resulting from falls. Raising awareness of these risks is essential for promoting safety and injury prevention, underscoring the importance of exercising caution and taking precautionary measures when participating in activities that involve elevated positions, such as tree climbing.
